# Relationship between gene duplicability and diversifiability in the topology of biochemical networks

**DOI:** 10.1186/1471-2164-15-577

**Published:** 2014-07-08

**Authors:** Zhanyong Guo, Wen Jiang, Nuno Lages, Wade Borcherds, Degeng Wang

**Affiliations:** Greehey Children’s Cancer Research Institute, University of Texas Health Science Center at San Antonio, 8403 Floyd Curl Drive, San Antonio, TX 78229-3900 USA; Department of Epidemiology and Biostatistics, University of Texas Health Science Center at San Antonio, 8403 Floyd Curl Drive, San Antonio, TX 78229-3900 USA; Department of Cell Biology, Microbiology and Molecular Biology, University of South Florida, 4202 E. Fowler Avenue, BSF218, Tampa, FL 33620 USA

## Abstract

**Background:**

Selective gene duplicability, the extensive expansion of a small number of gene families, is universal. Quantitatively, the number of genes (P_(K)_) with K duplicates in a genome decreases precipitously as K increases, and often follows a power law (P_(k)_∝k^-α^). Functional diversification, either neo- or sub-functionalization, is a major evolution route for duplicate genes.

**Results:**

Using three lines of genomic datasets, we studied the relationship between gene duplicability and diversifiability in the topology of biochemical networks. First, we explored scenario where two pathways in the biochemical networks antagonize each other. Synthetic knockout of respective genes for the two pathways rescues the phenotypic defects of each individual knockout. We identified duplicate gene pairs with sufficient divergences that represent this antagonism relationship in the yeast *S. cerevisiae*. Such pairs overwhelmingly belong to large gene families, thus tend to have high duplicability. Second, we used distances between proteins of duplicate genes in the protein interaction network as a metric of their diversification. The higher a gene’s duplicate count, the further the proteins of this gene and its duplicates drift away from one another in the networks, which is especially true for genetically antagonizing duplicate genes. Third, we computed a sequence-homology-based clustering coefficient to quantify sequence diversifiability among duplicate genes – the lower the coefficient, the more the sequences have diverged. Duplicate count (K) of a gene is negatively correlated to the clustering coefficient of its duplicates, suggesting that gene duplicability is related to the extent of sequence divergence within the duplicate gene family.

**Conclusion:**

Thus, a positive correlation exists between gene diversifiability and duplicability in the context of biochemical networks – an improvement of our understanding of gene duplicability.

## Background

Biochemical networks underlie essentially all cellular functions [1, 2]. Proteins do not act alone. Instead, they connect with each other to form pathways, such as the MAP kinase cascades and the glycolysis pathway. The connections are often direct physical protein-protein interactions or enzyme-substrate relationships. They can also be indirect ones. For instance, metabolic enzymes are usually connected through a chain of biochemical reactions they catalyze, even though the enzymes may not be physically associated with each other. And pathways in turn join together to form networks, such as the signaling and the metabolic networks. It is via such networks that genomic information gives rise to cellular functions and genotypes are translated into phenotypes. Biochemical network models have thus long served effectively as platforms for analysis of high-throughput experimental data, e.g., microarray or next generation sequencing based gene expression data [3–5].

A prominent category of constituents in biochemical networks is proteins encoded by duplicate genes, also termed paralogs [6]. Duplicate genes arose from genomic duplication events, which can be whole-genome duplication (WGD) or small-scale duplication (SSD). Genomic duplication is a major driving force of biological evolution [6–8]. Proteins of duplicate genes are thus abundant in biochemical networks. Moreover, their abundance increases along with genomic complexity, which is quantified by genome size, gene number, abundance of spliceosomal introns and mobile genetic elements, from bacterial to uni-cellular eukaryotes, to multi-cellular species [9]. Proteins of duplicate genes function and evolve in biochemical networks [10, 11]. Duplicate gene evolution is frequently analyzed in the context of biochemical networks, such as the protein-protein interaction networks [12–14] and the metabolic networks [15, 16], as well as other biological networks [17, 18].

A critical issue is gene duplicability. This term captures the selective gene duplication pattern universally observed in sequenced genomes [19–22]. A small portion of the genes in a genome has extraordinarily high duplicate counts, while the vast majority either are singletons or has only a few duplicates. In other words, a small number of gene families are selectively expanded during the genomic evolution process. Quantitatively, this phenomenon is often described by a power-law relationship between the number of genes (P_(K)_) with K duplicates and the duplicate count K, P_(k)_ ∝ k^-α^, with α as a positive constant. This relationship holds true regardless of which duplicate gene detection methods were used; FASTA, BLAST, as well as protein domain based methods have all been used [19, 22–24]. Moreover, this relationship holds true in bacterial, unicellular eukaryotic and multicellular genomes, and changes in the value of α can be used to quantify enrichment of duplicate genes as genomic complexity increases [22]. We operationally define gene duplicability, as popularly done, as the number of duplicates a gene has or the size of the gene family in a genome [25–28], although slightly different definitions also exist [29].

How and why did the selective gene duplicability pattern described above emerge? Two seemingly contradictive factors should contribute significantly: the opportunity to derive novel genetic materials from existing ones and the need to minimize deleterious effects of gene duplication. The first is the evolutionary advantage that genomic duplication confers to a species. A gene in the duplicated regions would have two copies. Subsequently, the pair of duplicate genes would accumulate mutations. Very often, one of the two duplicates formed a pseudo-gene, and became silenced [6, 30]. More importantly, the mutations sometimes led to functional diversification, either neo- or sub-functionalization, between the pair [7, 22, 31, 32]. This divergence can be in spatial-temporal expression patterns, interaction partners, enzymatic specificities of their proteins or subcellular locations of their proteins, etc. On the other hand, gene duplicability is limited, as postulated by the gene balance hypothesis, by the second factor – the potential detrimental effects of gene duplication due to disruption of the stoichiometric balance between protein products of duplicated and non-duplicated genes [28, 33, 34]. For instance, specific ratios among subunits are required for formation of protein complexes, which are major components of biochemical networks. Unless the genes for every subunit are all duplicated, a genomic duplication event would disrupt the balance. Rapid neo- or sub-functionalization between the two duplicates would restore the stoichiometric balance and alleviate this gene dosage constrain, thus enhancing gene duplicability. For instance, in multi-cellular genomes, enhanced functional diversification through accumulation of introns has been associated with higher duplicate gene survival rates [9, 35].

Thus, functional diversification of duplicate genes not only promotes genomic functional innovation, but also alleviates potential deleterious effect of gene duplication. It is very likely that selective gene family expansion and enhanced diversification within the expanding families proceeded inextricably hand-in-hand. In other words, duplicate genes in larger gene families should have diverged from each other to a higher extent than those in smaller families. For the sake of consistency with the usage of “duplicability” to refer to the propensity of a gene to be duplicated (duplication rate and duplicate survivability) [27], we use the term “diversifiability” as its sister term to refer to the propensity of duplicate genes to undergo diversification (neo- or sub-functionalization). Similar to duplicability being operationally computed as the number of duplicate a gene has or the size of the duplicate gene family, diversifiability can be computed as the degree of diversification among duplicate genes. We hypothesized positive correlations between gene duplicability and diversifiability.

Testing the hypothesis requires quantifying diversifiability of duplicate genes. Three metrics were used in this study. Two of them were developed in the context of biochemical network; one measures the extent to which duplicate genes diverge sufficiently for their proteins to participate in mutually antagonizing pathways in a network, the other the pair-wise shortest network distance among the proteins of duplicate genes. As the third metric, a protein sequence homology based clustering coefficient was used to quantify sequence divergence among duplicate genes. We report, for each of the three metrics, positive correlation between gene duplicability and diversifiability.

## Results

### Quantification of gene duplicability

**Figure 1:**
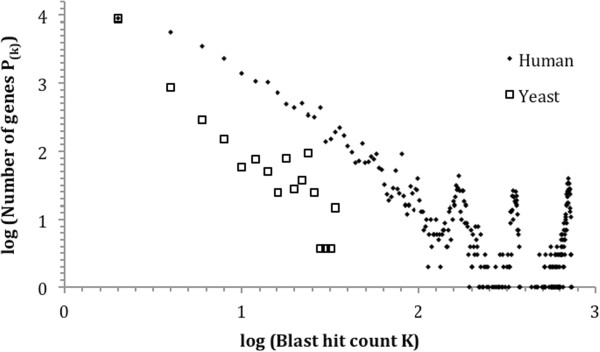
**Log-log plot of the numbers of protein-coding genes P**_**(K)**_ **with duplicability K vs. K in the yeast** ***S. cerevisiae*** **and human.** As described in Materials and Methods, duplicability K of a gene was calculated as BLAST hit count of its protein in an all-against-all BLAST, with a threshold BLAST E-value of 10^−30^. Linear relationships were observed, indicating power-law relationship between P_(K)_ and K (P_(K)_ ∝ K^-α^). And the slopes of the linear relationships – that is, the α values – were different between yeast and human. To better illustrate this difference, *S. cerevisiae* data points were shifted upward to overlap the leftmost data points of the two species.

As the goal of this study is to detect potential relationship between gene duplicability and diversifiability, it is necessary to measure gene duplicability. We performed respective all-against-all BLAST for protein sequences encoded in the yeast *S. cerevisiae* and the human genome, with a threshold E-value of 10^−30^. BLAST hit count (K) was calculated for each protein. We used the value of K as a quantifier of duplicability of the corresponding gene – the higher the value of K, the higher the duplicability. As previously done for the *S. cerevisiae* and the *C. elegans* proteomes [22], we created the log-log plot of the number of proteins with K BLAST hits (P_(K)_) vs. K for the human proteome. A linear relationship between log(P_(K)_) and log(K) was observed (Figure 1). This indicates a power-law relationship – P_(k)_ ∝ k^-α^ with the exponent constant α being the slope of the linear relationship. Moreover, the decrease of log(P_(K)_) as log(K) increased was slower in the human proteome than in the yeast proteome, i.e., a lower α value of the power-law relationship, reflecting higher duplicate gene abundance in multicellular genome.

To put it another way, we used all-against-all BLAST results of a proteome to cast the proteins into a weighted sequence homology network for that species. Nodes and edges of the network were proteins and pair-wise protein homology relationship, respectively. Edges were weighted by the strength of the homology relationship, as quantified by BLAST output parameters such as the E-value. Connectivity of proteins, a key parameter in network analysis, equals to the values of their BLAST hit count K, which, as described above, follows a power-law distribution. The network is thus scale-free. This line of analysis, to be discussed later, led us to effectively adopt another standard network analysis parameter in this study.

Our question then became whether the value of K is correlated with gene diversifiability, the extent to which these duplicate genes have diverged. Thus, the next step was to evaluate duplicate gene diversifiability, which we performed in the context of biochemical networks.

### Pairs of duplicate genes that have diverged to mutual genetic antagonism tend to belong to high duplicability gene families

We looked for an approach to identify cases of high diversifiability among duplicate genes, so that we could then determine whether high diversifiability is associated with high gene duplicability, i.e., high K values. We took advantage of the observation that two proteins may participate in pathways that antagonize each other in a biochemical network. Genetically, synthetic knockout of both of their genes rescues or alleviates the phenotypic defects caused by the individual knockout of either one. Pairs of duplicate genes that exhibit this genetic antagonism relationship must have gone through a switch from their initial identical functions upon gene duplication to functional antagonism – a complete functional diversification process. Such pairs are thus perfect examples of high functional diversifiability. For instance, the *S. cerevisiae* Pif1 and Rrm3 DNA helicases share high sequence homology (BLAST E-value 2E-103), but they have opposite effects on ribosome DNA replication. Pif1 enhances necessary pausing, whereas Rrm3 promotes continuous progression of the replication forks [36]. Moreover, synthetic knockout has been systematically carried out in the yeast *S. cerevisiae*, making it possible to identify pairs of mutually antagonizing duplicate genes. We thus identified, as described in Materials and Methods, all such *S. cerevisiae* duplicate gene pairs from the SGD database. As a control for our analysis, we also identified pairs of duplicate genes that exhibit the opposite relationship – mutual genetic complement. In such relationships, synthetic knockout of both genes causes more severe phenotypic defects than each of the two individual knockouts. The two duplicate genes in such pairs retain functional similarity, and are often functionally interchangeable. The two groups of duplicate gene pairs gave us an opportunity to determine whether high diversifiability is accompanied by high K values, and thus enhanced duplicability.

**Figure 2:**
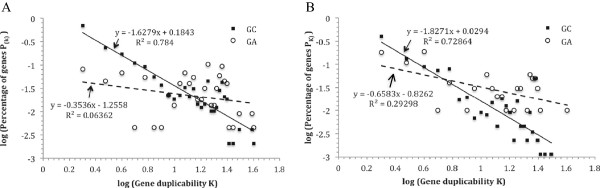
**Comparison of duplicability (K) distributions within the two groups of duplicate genes: genes in genetically antagonizing (GA) duplicate pairs (dashed line and white circle) vs. those in genetically complementing (GC) pairs (Solid line and black square). A**: log(P_(K)_) *vs.* log(K) plot of genes whose BLAST hits enclose both proteins of a corresponding duplicate gene pair. **B**: log(P_(K)_) *vs.* log(K) plot of genes in corresponding group of duplicate gene pairs. The gene pairs were identified as described in Materials and Methods. The horizontal axis is the logarithms of gene duplicability K, which, as described in Materials and Methods, was calculated as BLAST hit count of a gene’s protein. Vertical axis is the logarithms of P_(K)_. Linear regression lines, regression equations and R^2^ values of the regression are shown. In both panels, the GC gene data (black squares) fit well into power-law relationships, whereas the GA gene data (white circles) fit, if at all, poorly.

We first assessed whether genetically antagonizing (GA) duplicate gene pairs were more likely to belong to larger duplicate gene families than genetically complementing (GC) pairs. The approach was to collect, for each of the two groups of duplicate gene pairs, the set of genes whose BLAST hits enclose the proteins of both genes in a pair. We then determined which of the two sets of identified genes have higher K values, i.e., whether two mutually antagonizing or complementing duplicate genes tend to have their proteins co-occur in BLAST hits of genes with higher K values. The results are illustrated in the form of log-log plots in Figure 2A. The vertical axis represents the logarithms of percentage of genes, and the horizontal axis the logarithms of K values. A clear linear decay fit the log-log data well (with a R^2^ value of 0.78) in the case of GC pairs. The α value of the power-law relationship was 1.63. The log-log data of the GA duplicate gene pairs, on the other hand, fit very poorly (with a R^2^ value of 0.06) into a linear decay relationship. The power-law relationship, if it was at all, had a much lower α value of 0.35, indicating much slower decrease of the count of gene pairs as K increases. Thus, proteins of pairs of antagonizing duplicate genes tend to be grouped together in BLAST hits of the proteins of high duplicability genes. In other words, GC duplicate gene pairs tend to be associated with low duplicability genes and smaller gene families, whereas GA pairs are much more likely to be associated with high duplicability genes and larger gene families.

**Figure 3:**
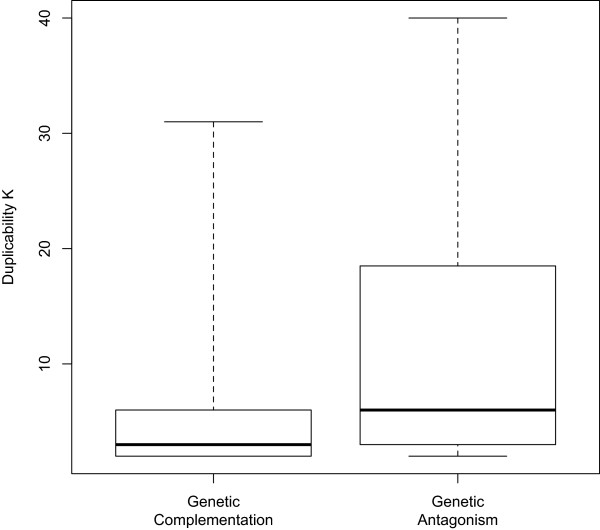
**Box plot of duplicability K of duplicate genes involved in mutual genetic antagonism (GA) or genetic complementation (GC) relationships.** GA and GC relationships between a pair of duplicate genes were identified as described in Materials and Methods. Duplicate genes involved in GA relationship exhibit a higher median K value, and their K values display an overall shift toward higher value range.

We also determined whether genes in GA duplicate gene pairs themselves tend to have higher K values. The approach was to compare distributions of K values of the genes involved in the two groups of duplicate gene pairs. We calculated the P_(K)_ vs. K distribution for each of the two groups. Figures 2B displays the distributions for the GA and GC groups, respectively. Once again, a clear power-law decay, and thus scale-free relationship, was found in the case of GC pairs, and a power regression fit the data nicely, with a R^2^ value of 0.73, into a power-law relationship with a α value of 1.83. As for GA pairs, the data fit poorly (with a R^2^ value of 0.29), if at all, into a power law relationship; and the value of α, 0.66, is much lower. The lower α value indicates slower P_(K)_ decay as K increases. This leads to, as shown in a boxplot in Figure 3, a higher median K value and a shift toward a higher K value range. Thus, genes in GA duplicate gene pairs tend to have higher K values, and thus higher duplicability.

Thus, the two genes in GA duplicate gene pairs tend to have their proteins co-occur in BLAST hits of genes with high K values, and they themselves also tend to have higher K values. The results strongly suggest that the higher the duplicability, the higher the functional diversifiability becomes. Genes in smaller gene families tend to be less diversified, so their functions are more likely to compensate each other, giving rise to genetic redundancy and robustness. Genes in larger families, on the other hand, are more likely to have neo- and/or sub-functionalized more to assume different, or even antagonizing, functions. Thus, their functions are less likely to compensate each other. Instead, they contribute to evolutionary functional innovation.

### Proteins of genes from high duplicability families tend to be farther away from each other in the protein-protein interaction networks

For a more direct quantifier of functional diversifiability in the context of biochemical networks, we evaluated pair-wise network distances among proteins. Since they diverge from the same ancestor, duplicate gene pairs are expected to be more functionally related than non-duplicate pairs in the topology of biochemical networks. Prior to functional diversification, their proteins shared the same set of interaction partners. During subsequent evolutionary network re-wiring, the proteins went though various levels of functional diversification and switched interaction partners. Very often, these diverging pairs eventually lost all common interaction partners, although they are still more likely than expected by random chance to participate in the same network domains, i.e., functional modules. We tested whether protein-to-protein network distances were a reflection of overall functional similarity among duplicate genes; that is, besides genetic antagonism, whether network distance could be used as another quantifier of functional diversifiability to study relationship between duplicability and functional diversifiability of genes.

#### Proteins of duplicate gene pairs tend to be closer to each other in protein-protein interaction networks

**Figure 4:**
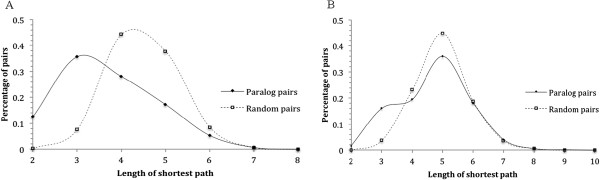
**Comparison of the distributions of pair-wise network distances between pairs of paralogous protein pairs vs. that between non-paralogous protein pairs in the protein-protein interaction networks of the yeast** ***S. cerevisiae*** **(A) and human (B).** Same number of non-paralogous protein pairs as paralogous pairs were randomly picked from the networks. The horizontal axis is the network distance, calculated as the length of shortest path between two proteins in the protein-protein interaction network (in terms of number of proteins in the path; 2 indicates directly connected nodes). The vertical axis is the percentage of gene pairs with corresponding network distance. The p-value of the two distributions in **A** is 1.886e-7 (Pearson’s χ^2^ test). When the data in **B** were subdivided, based on the shape of the distributions, into two groups using a boundary of 3.5, the two distributions have a p-value of 0.003 (Pearson’s χ^2^ test).

To test whether functional similarity between duplicate genes was reflected in the network distances between their proteins, we calculated network distance between proteins of each pair of duplicate genes in the *S. cerevisiae* network. As a control, we randomly picked the same number of non-duplicate gene pairs from the network, and calculated network distance between the two proteins in each of them. A comparison of the two sets of network distances is shown in Figure 4A. For pairs of duplicate genes, a significant portion of their proteins were found directly connected with each other in this analysis; the distribution of network distances between their proteins peaked at 3. As for the randomly picked pairs of non-duplicate genes, on the other hand, very few of their proteins were found to be directly connected, and the distribution of network distances between their proteins peaked at 4. The p-value of the two distributions is 1.886e-7 (Pearson’s χ^2^ test). Thus, proteins of a pair of duplicate genes tend to be closer to each other than those of a pair of non-duplicate genes in the *S. cerevisiae* network.

We tested whether this observation in *S. cerevisiae* remained true in the human network. As described in Materials and Methods, we downloaded human protein interaction data from the IntAct database [37]. We collected all pairs of proteins of duplicate genes and calculated the distance in the network for each of them. Once again, we randomly picked the same number of pairs of non-paralogous proteins from the network and calculated distance for each of them. A comparison of the two sets of network distances is shown in Figure 4b. The distribution of network distances of randomly picked non-paralogous pairs resembled a normal distribution, with a single peak at network distance 5. The network distances between paralogous proteins, on the other hand, exhibited a very different distribution. The distribution has a similar peak at the network distance 5, but a significant portion of the distances shifted leftward, leading to a shoulder in the short network distance region of the distribution. Consequently, for 17.7% of duplicate gene pairs, their proteins were observed to be close to each other in the network, with a network distance of 2 or 3, whereas the percentage was only 3.9% for randomly picked non-duplicate pairs. When the data are subdivided into two groups using a boundary of 3.5, the two distributions have a p-value of 0.003 (Pearson’s χ^2^ test).

#### Proteins of genetically antagonizing (GA) duplicate gene pairs tend to have longer network distances than those of genetically complementing (GC) pairs

The result suggests that, as a reflection of their functional similarity, paralogous proteins have an overall tendency to be closer to one another in both the human and the yeast biochemical networks. However, different duplicate gene pairs might have different levels of functional similarity. Also, not all pairs of duplicate genes retain high functional similarity during genomic evolution, as evolution pressure is often for neo- and/or sub-functionalization. We tested whether the network distance tends to be longer between the two proteins of a highly diverged pair of duplicate genes.

**Figure 5:**
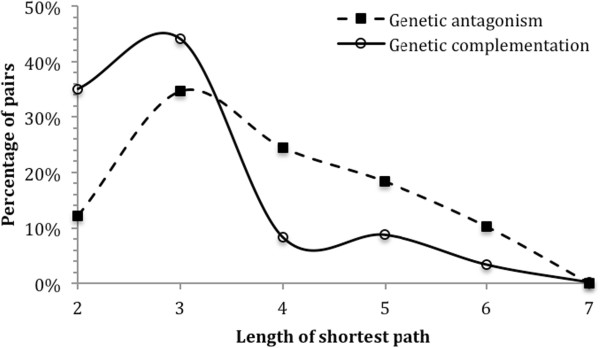
**Comparison of the distributions of pair-wise protein-protein network distances within the two groups of duplicate gene pairs: genetic antagonism (GA) vs. genetic complementation (GC).** The horizontal axis is the network distance, calculated as the length of shortest path between two proteins in the protein-protein interaction network of the yeast *S. cerevisiae*. The vertical axis is the percentage of gene pairs with corresponding network distance. The p-value of the two distributions is 5.6e-05 (Pearson’s χ^2^ test).

Once again, the set of GA duplicate gene pairs in *S. cerevisiae* was used, with the set of GC pairs serving as a control. For each duplicate gene pair in each of the two groups, we determined the network distance between their proteins. Figure 5 shows the distributions of the GA and the GC gene pairs in relation to the network distance. The number of nodes in the path quantifies the path length. The minimum length of 2 indicates directly connected nodes. GC pairs concentrated in a shorter path length range, while GA pairs were more likely to have a longer shortest path. The p-value of the two distributions is 5.6e-05 (Pearson’s χ^2^ test). In a word, proteins of those duplicate gene pairs that have fully diversified into genetic antagonism tend to have longer network distances from each other.

#### Network distances between proteins of genes from high duplicability families tend to be longer

**Figure 6:**
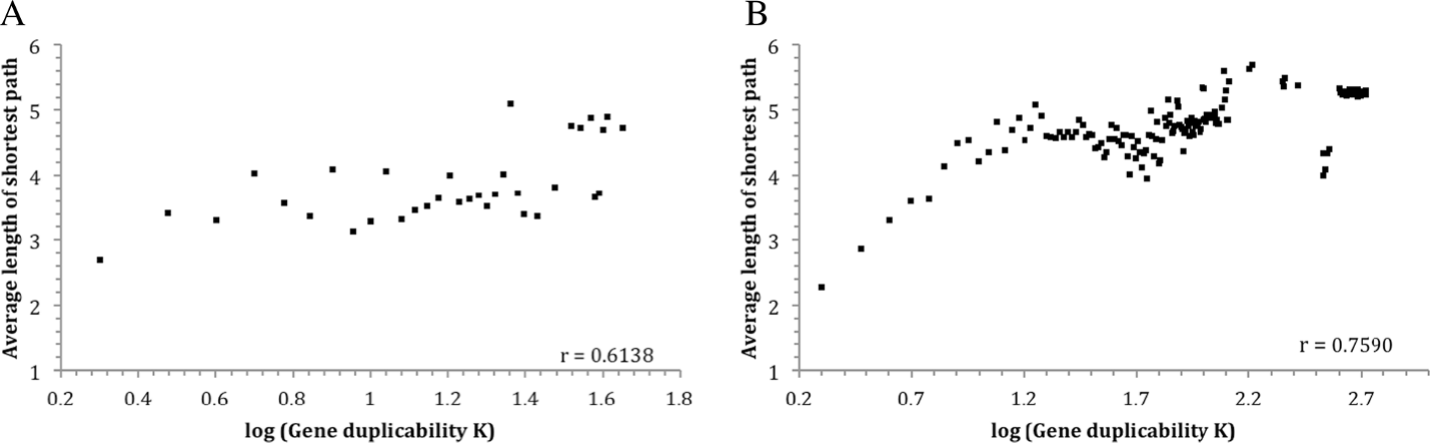
**Relationship between gene duplicability (K) and average network distance among proteins of corresponding duplicate genes in the yeast** ***S. cerevisiae*** **(A) and human (B) protein-protein interaction networks.** Gene duplicability, as described in Materials and Methods, was calculated as BLAST hit count of the gene’s protein. Average network distances were calculated as the average length of all pair-wise shortest paths among the proteins of the gene and its duplicates. The data were binned with a bin-size of 1 according to gene duplicability K. The vertical axis is the average network distance within each bin.

As discussed earlier (Figures 2 and 3), pairs of GA duplicate genes tend to belong to large gene families. Two genes in such pairs tend to co-occur in duplicate lists of genes with higher K values, and they themselves have higher duplicability (K) values. Thus, both high duplicability and longer network distances between their proteins are associated with enhanced diversifiability of mutually antagonizing duplicate gene pairs. We tested whether this observation can be generalized to the whole genome, i.e., the higher a gene’s duplicability K, the longer the pair-wise network distances among the proteins of this gene and its duplicates tend to be. As shown in Figure 6, this is indeed true. Average network distances among the proteins of a gene and its duplicates have a positive correlation with the K value of the gene. The two have a correlation coefficient of 0.61 in yeast (Figure 6A), and 0.76 in human (Figure 6B).

These results suggest that network distance can be used as a quantifier of duplicate gene diversifiability in the topology of biochemical networks; longer distances imply higher diversifiability. Functional diversifiability measured with this parameter correlates positively with gene duplicability. Thus, network distance and genetic relationship provide two lines of evidence that enhanced functional diversifiability accompanied hand-in-hand enhanced gene duplicability.

### The positive correlation between duplicability and diversifiability applies to both whole-genome duplicate (WGD) and small-scale duplication (SSD) duplicate genes

**Figure 7:**
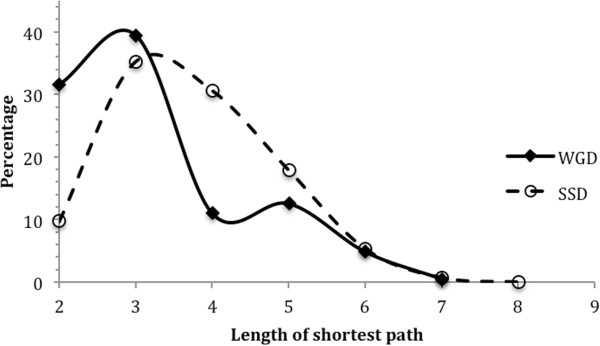
**Comparison of network distances between the proteins of whole-genome duplication (WGD) and small-scale duplication (SSD) duplicate gene pairs.** The horizontal axis is the length of shortest network path (network distance) between the two proteins of a duplicate gene pair. The vertical axis is the percentage of pairs with the corresponding network distance. SSD pairs tend to have longer distances. The two distributions have a p-value of 0.001 (Pearson’s χ^2^ test).

WGD duplicate genes, unlike SSD duplicate genes, maintained the stoichiometric ratio between their proteins, circumventing the gene dosage evolutionary constraint and thus have higher retention rates. They also diverge slower than SSD duplicate genes, as they are under pressure to preserve the stoichiometric ratio [38–41]. As shown in Table 1 and Figure 7, this is reflected in their genetic relationship and the network distances between their proteins. WGD duplicate gene pairs are much more likely to genetically complement each other; 41.7 percent (194 out of 465) of WGD pairs, whereas only 6.7 percent (251 out of 3762) of SSD pairs, have such relationship. The ratio of number of genetically complementing pairs to that of antagonizing pairs is 21.6:1 (194 to 9) for WGD, and only 5.8:1 (251 to 43) for SSD (Table 1). Additionally, proteins of WGD pairs have overall shorter network distances than those of SSD pairs. The distances are 3 or less for a majority (>70%) of WGD pairs, but are 4 or longer for ~55% of SSD pairs (Figure 7). The two distributions have a p-value of 0.001 (Pearson’s χ^2^ test).

**Table 1:** **Respective distribution of GC and GA pairs, and their ratio, in WGD and SSD duplicate gene pairs**

	Pair count	GC (%)	GA (%)	GC/GA Ratio
WGD	465	194 (41.7)	9 (1.9)	21.6
SSD	3762	251 (6.7)	43 (1.1)	5.8

**Figure 8:**
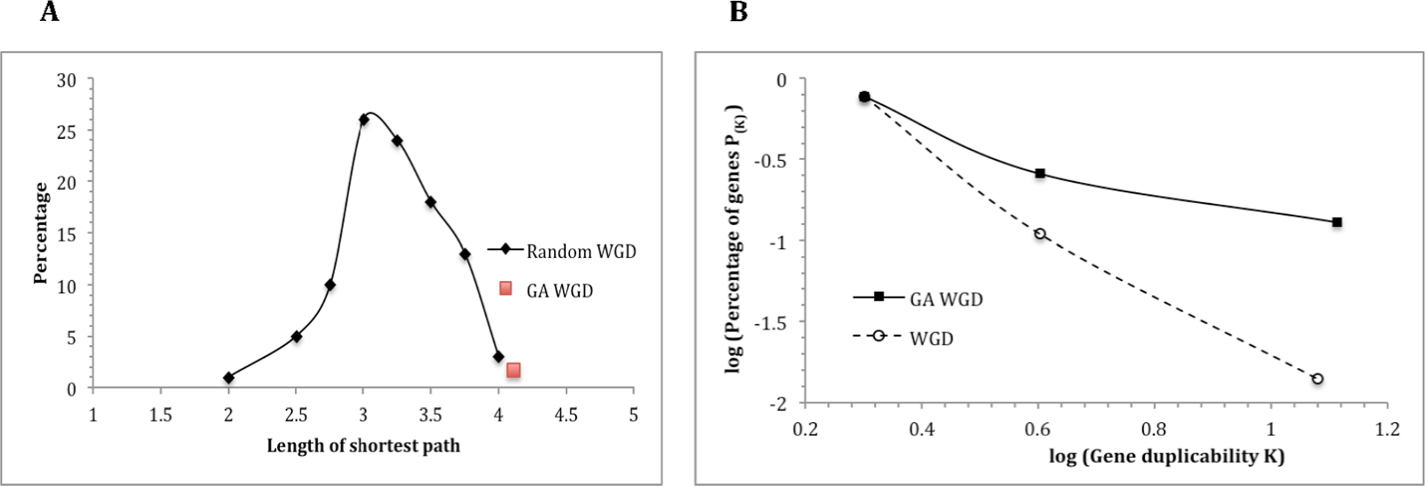
**Comparison of protein-to-protein network distances (A) and gene duplicability (B) of genetically antagonizing (GA) WGD duplicate gene pairs and those of the rest of WGD pairs. A**: average network distance of the 9 GA WGD duplicate gene pairs (red square) compared with histogram/distribution of the averages for groups of random WGD pairs (the curve). 100 groups, each consisting of 9 randomly selected WGD pairs as described in the text, were used to generate the histogram. Horizontal axis is the average length of shortest network paths (network distance). Vertical axis is the percentage of the groups with the corresponding average network distance. The vertical position of the average for the 9 GA pairs was based on its p-value (0.017). **B**: log(P_(K)_) *vs.* log(K) plots of genes in GA WGD duplicate gene pairs and of genes in all WGD pairs. Gene duplicability K was calculated as BLAST hit counts. To better illustrate the difference, the GA data points were shifted upward to overlap the leftmost data points of the two plots.

We also compared the 9 genetically antagonizing (GA) WGD pairs with the rest of the WGD pairs (Figure 8). We randomly selected 9 WGD pairs and calculated the average network distances between their proteins. This process was repeated 100 times. We then compared the distribution of the 100 average network distances and the average distances of the 9 GA pairs. The comparison showed that the 9 GA pairs have longer distances than randomly selected pairs, with a p-value of 0.017 (Figure 8A). Additionally, we compared the log(P_(K)_)-log(K) curve of the 18 genes in the 9 GA WGD pairs and that of the rest of the WGD duplicate genes (Figure 8B). The 18 genes exhibit 3 K values and form a 3-data-point curve. To make the curve for the rest of the WGD genes, we used only the genes with one of the three K values; this ensured that the two log-log curves are equivalent and comparable. Log(P_(K)_) decreases as log(K) increases in both curves, but the decrease is much slower for the 18 genes, suggesting enhanced duplicability.

Thus, the positive correlation between gene duplicability and diversifiability applies to both WGD and SSD duplicate genes. We next examined whether the enhanced functional diversifiability observed among high duplicability genes is accompanied by enhanced diversification at the sequence level, as sequence is the primary determinant of protein functions.

### Genes from high duplicability families tend to be more divergent at the sequence level

To quantify sequence diversification among a set of duplicate genes, we took advantage of a standard parameter in network analysis – the clustering coefficient. As discussed earlier, our approach for duplicate gene identification essentially cast the proteins of a proteome into a sequence homology network, where the nodes are sequences and the edges represent pair-wise homology relationship identified in the all-against-all BLAST result. Weight of each edge was the strength of the sequence homology relationship as quantified by the pair-wise BLAST E-value – the lower the E-value, the stronger the homology relationship. Thus the networks are weighted. The power-law relationship between P_(K)_ and K (P_(k)_ ∝ k^-α^) indicates the sequence homology networks are, as many real-world complex networks, scale free. The clustering coefficient is routinely used in analysis of such networks to quantify connectivity among immediate neighbors of a node [42], which in the context of our sequence homology network would be homology among duplicates of a gene. Thus, we used this parameter to quantify sequence diversifiability among a gene’s duplicates – the lower the coefficient, the higher the extent to which these sequences have diverged. To calculate the weighted clustering coefficient (C), we used equation 1 [43] below:
1

Where K_i_ is the duplicability (connectivity in the homology network) of protein i; proteins j and k represent any pair of immediate neighbors of protein i. *W*_ij_ is the weight of the edge between proteins i and j (0 < *W* ≤ 1), which is calculated as the negative logarithm of the pair-wise BLAST E-value normalized by the maximum E-value of each individual clusters. In short, C_i_ indicates the probability for two immediate neighbors of node i to form a non-zero weighted triangle together with i.

**Figure 9:**
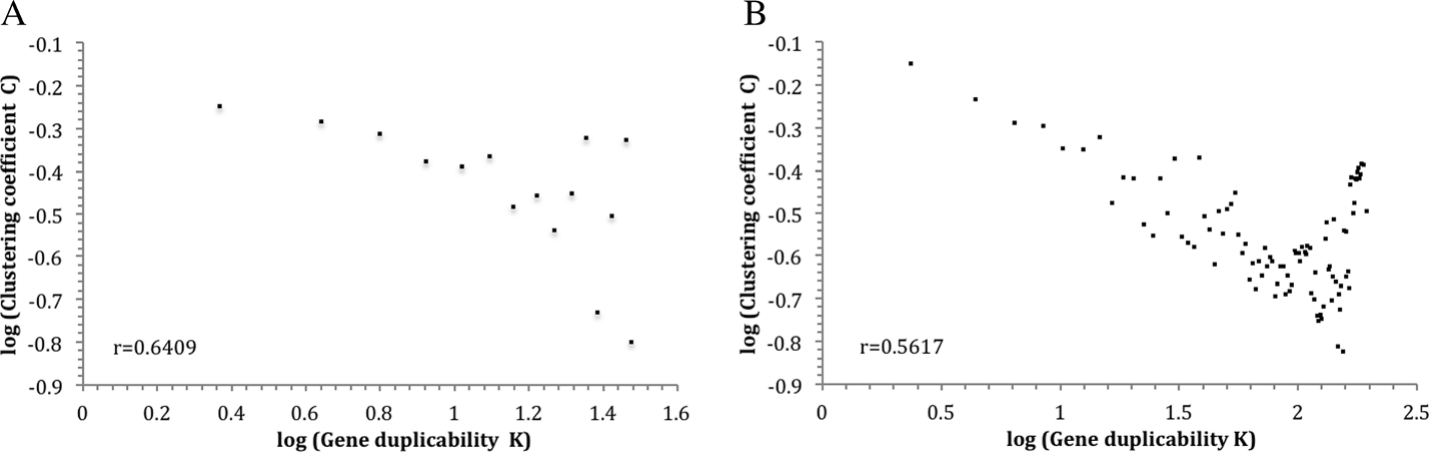
**Log-log plot of gene duplicability K** ***vs.*** **clustering coefficient C in the yeast** ***S. cerevisiae*** **(A) and human (B).** Gene duplicability K, as described in Materials and Methods, was calculated as BLAST hit count of the gene’s protein. Clustering coefficient C was calculated using equation 1 described in text. Maximal BLAST E-value of individual cluster was used in weighted clustering coefficient calculation. The data were binned with a bine-size of 2 according to gene duplicability K.

We calculated, for each gene in the *S. cerevisiae* genome, the value of C. We then tested whether C is correlated with the gene’s duplicability K. In real world scale-free networks, the two parameters are usually negatively correlated. As shown in Figure 9A, this is also true in the yeast protein sequence homology network. When a gene has a high K value, the C value among its duplicates tends to be low. The log(C) vs. log(K) plot displays some degree of linearity, with a Pearson correlation coefficient of −0.64 and a p-value of 0.005. Thus, higher gene duplicability is accompanied by higher sequence diversifiability.

We also examined the relationship between C and K for human genes. The result is shown in Figure 9B. Once again, a negative correlation is observed. The log(C) vs. log(K) plot displays a good linear relationship, with a Pearson correlation coefficient of −0.56 and a p-value of 1.59e-09. Thus, the positive correlation between gene duplicability and diversifiability is true in human as well.

Taken together, these results from *S. cerevisiae* and human suggest that higher gene duplicability is accompanied by enhanced diversifiability at the sequence level, the 3^rd^ line of evidence that high gene duplicability and diversifiability acted hand-in-hand during selective gene family expansion in genomic evolution. Thus, this study has used three parameters, each measuring one aspect of duplicate gene diversifiability. Results from all of them support the notion that diversifiability is an important determinant of gene duplicability in evolution.

## Discussion

Selective gene duplicability is universally observed in all sequenced genomes [21]. Gene duplication is a major source of genetic material for functional innovation in evolution, leading to a high genomic abundance of duplicate genes [6–8]. On the other hand, in a genome, most protein-coding duplicate genes belong to a small number of gene families while genes outside these large families have few or no duplicates, i.e., a small number of gene/protein families are selectively and extensively expanded. Quantitatively, the number of genes with K duplicates (P_(K)_) often follows a power law, i.e., P_(K)_ ∝ K^-α^, with α as a positive constant [19, 22, 23]. As K increases, P_(K)_ decreases precipitously. High duplicability is confined to select groups of genes. This study demonstrates, at the genomic level, that this elevated gene duplicability is associated with higher degree of functional and sequence diversification. We use the term gene diversifiability as a sister term of gene duplicability to describe the degree of diversification among duplicate genes. In a word, high gene duplicability and high gene diversifiability acted side-by-side to promote functional innovation during evolution. This conclusion is supported, as discussed below, by results from each of the three diversifiability measurement methods.

First, we took advantage of systematic genetic interaction data available for the yeast *S. cerevisiae*, and identified all pairs of genetically antagonizing duplicate genes as representatives of high diversifiability between duplicate genes. For instance, as mentioned earlier, the homologous DNA helicases Pif1 and Rrm3 exert opposite effects on ribosome and mitochondria DNA replication [36, 44], and the protein kinases FUS3 and CDC28 (E-value 9E-46) have counteractive control over cell polarization during mating [45]. Genes in such fully diversified pairs, we found, overwhelmingly belong to large duplicate gene groups – they have higher duplicate counts (K) and thus higher duplicability. As a result, the relationship between the number of proteins (P_(K)_) with K duplicates and the duplicate count K among these genes deviates significantly from a power-law relationship. These results show that high diversifiability genes tend to have high duplicability as well.

Second, we examined network distances (shortest path) between proteins of duplicate genes in the protein-protein interaction network as a metric of their diversifiability. In both human and yeast, the higher a gene’s duplicate count, the further its duplicates’ protein products tend to drift away from its own protein (longer shortest path) in the networks. In yeast, proteins of genetically antagonizing duplicate gene pairs tend to have longer network distances than those of genetically complementing pairs. This further confirms that gene diversifiability is positively correlated to gene duplicability.

Third, we measured sequence divergence within duplicate gene groups, using a homology-based clustering coefficient, which increases inversely to sequence divergence. A negative correlation was observed between duplicability K of a gene and the clustering coefficient among its duplicates. Thus, once again, gene duplicability is positively correlated to gene diversifiability.

Taken together, these results demonstrate enhanced diversifiability among genes in large duplicate gene families. Current knowledge suggests that this enhanced diversifiability played two roles in duplicate gene evolution – functional innovation and, at the same time, alleviation of the gene-dosage evolution constrain. As discussed in the introduction, cellular processes usually consist of the actions of multiple proteins and require specific stoichiometry ratios among the proteins. Gene duplication breaks this balance between proteins of duplicated and non-duplicated genes [34]. Thus, without functional diversification, duplicate genes not only confer no evolution advantage (functional innovation), but also are also potentially deleterious. Very often, one of the two copies of the gene disappears in order for the gene balance to be restored during subsequent evolution [30]. When both survive, they must quickly neo- or sub-functionalize, both to alleviate the gene-dosage constrain and to meet the evolution demand for functional innovation [46, 47]. Thus, enhanced diversifiability must have accompanied selective expansion of gene families during genomic evolution. And, in addition to duplication rates, gene duplicability is also determined by survival rates of duplicate genes.

Our results are consistent with the observations by Lynch and Conery that duplicate gene survival rate increases from prokaryotes to multicellular eukaryotes [9]. It is obvious that additional venues for functional diversifiability of duplicate genes became available as cellular and organismal complexity increases from prokaryotes to eukaryotes, and on to multicellular species. For instance, eukaryotic cells are compartmentalized, making subcellular localization a potential venue for gene diversification. Multi-cellular species provide an additional layer of functional diversification, diversifying cell/tissue distribution patterns. The evolutionary pressure is to create complementary expression patterns among duplicate genes [22, 32]. Protein products of duplicate genes often do not co-exist in the same cell or subcellular location. They can preserve their biochemical specificity, e.g. interacting with the same set of proteins, without breaking the gene dosage balance. The gene dosage constraint is thus lessened, explaining the higher retention rate of duplicate genes observed in multi-cellular genomes.

The higher duplicate gene retention rates due to enhanced diversifiability in turn leads to increases in duplicate gene abundance from prokaryotes to multicellular eukaryotes. This is consistent with changes in the exponent (α) values of the power-law relationship between P_(K)_ and K, P_(K)_ ∝ K^-α^. The value of α is a duplicate protein coding gene abundance quantifier [22]. Its value decreases from prokaryote to unicellular eukaryote, and to multicellular eukaryote. A lower value of α indicates that P_(K)_ decreases at a slower pace as K increases, and therefore dictates higher paralog abundance. As discussed above, the eukaryotic cellular environment is more permissive for gene duplication, allowing duplicate genes to be partitioned to different cellular compartments to bypass the dosage evolutionary constraint. Moreover, in multicellular eukaryotes, duplicate genes can potentially overcome the dosage evolutionary constraint through expression in different cell types.

The permissive environment in multicellular eukaryotes enabled extensive expansion of many gene families. On the other hand, the expansion is often species-specific, such as the explosive expansion of the receptor serine/threonine kinase family and the receptor tyrosine kinase family in plants and animals, respectively [48, 49]. Species-specific factors enhancing diversifiability and duplicability within these gene families, and how their expansion contributed to evolutionary fitness of the specific species, will be an interesting research topic.

## Conclusions

In summary, we report three lines of evidence supporting a positive relationship between gene diversifiability and duplicability. The significance of this work can be illustrated through an analogy. Both genetic sequences and English literature are linear strings of alphabets [50, 51]. If the genome is the “book” of life, as it is often referred to, evolution is the “writer” of the book. The process of gene duplication and subsequent diversification is in turn intuitively analogous to the frequently used copy-paste-revise writing technique – copying and pasting texts from other sources, and then revising and merging them into current context. Gene duplication and fate of duplicate genes has thus been fundamental in genomics and evolution biology. This study improves our understanding of this critical process in the context of biochemical networks.

## Methods

### Sequence data and duplicate gene identification

Yeast (*S. cerevisiae*) proteome sequences were downloaded from the Saccharomyces Genome Database (SGD) [52]. Human (Homo sapiens) sequences were downloaded from NCBI. In yeast, protein sequences encoded by dubious ORF and transposable-element enclosed genes were removed. The yeast and human sequences were then used in respective all-against-all BLAST analysis to identify pairs of duplicate genes [53]. A stringent threshold BLAST E-value of 10^−30^ is used. In yeast, a total of 7,556 pairs, involving 1,945 genes, were identified. In human 99,611 pairs, involving 13,309 genes, were identified.

### Genetic antagonism (GA) and complementation (GC) data of the yeast *S. cerevisiae*

Synthetic knockout data was downloaded from the SGD database [52]. We identified all genetic interactions between duplicate genes, and assigned them to the (GA) or the (GC) categories based on SGD annotation. Genes were designated as antagonistic when their synthetic knockout rescues or alleviates the phenotypic defects caused by individual knockout of either one. Such genetic interactions were annotated as synthetic rescue or phenotypic suppression in the SGD dataset, and were categorized accordingly as GA in our analysis. The opposite, GC, means that synthetic knockout of two genes causing severer phenotypic defect than individual knockout. Such interactions were annotated as synthetic lethality or phenotypic enhancement in SGD, and were categorized accordingly as GC.

### Protein-protein interaction network data

Yeast protein interaction data were downloaded from the *Saccharomyces* Genome Database (SGD) [52]. The dataset contains 38573 interactions. Individual proteins have up to 332 interactions. Human protein interaction data was from the IntAct database [37]. The dataset contains 140,268 interactions. Individual proteins have up to 1,225 interactions.

### Shortest path analysis

Pair-wise shortest path in the protein interaction network was calculated between proteins using the depth-first search (DFS) algorithm. The length of a shortest path is calculated as the number of proteins in the path. For instance, a length of 2 indicates the two proteins directly connect with each other, and a length of 3 indicates that there is one protein between them. In analyzing the distribution of shortest paths between paralogous pairs, an equal number of non-paralogous pairs randomly picked from the network were used as a control.

### WGD and SSD data set

The whole-genome duplicate (WGD) data set is taken from The Yeast Gene Order Browser [54, 55]. Within the entire 548 pairs, 465 pairs (930 genes) still have BLAST E-values smaller than 1E-30 between their proteins and are included with our duplicate gene pair list. We use this dataset of 465 pairs for our WGD analysis. The rest (3762 pairs) of pairs in our list are considered as small-scale duplication (SSD) pairs. These 3762 pairs consist of 1441 genes. WGD and SSD pairs share 321 genes. As expected from the way they were identified, each WGD duplicate gene show up only once in the whole list of WGD pairs.
